# Comparative Study of the Accuracy of Different Techniques for the Laboratory Diagnosis of Schistosomiasis Mansoni in Areas of Low Endemicity in Barra Mansa City, Rio de Janeiro State, Brazil

**DOI:** 10.1155/2015/135689

**Published:** 2015-10-04

**Authors:** Maria Cristina Carvalho Espírito-Santo, Mónica Viviana Alvarado-Mora, Pedro Luiz Silva Pinto, Maria Carmen Arroyo Sanchez, Emmanuel Dias-Neto, Vera Lúcia Pagliusi Castilho, Elenice Messias do Nascimento Gonçalves, Pedro Paulo Chieffi, Expedito José de Albuquerque Luna, João Renato Rebello Pinho, Flair José Carrilho, Ronaldo Cesar Borges Gryschek

**Affiliations:** ^1^Department of Infectious and Parasitic Diseases and Laboratory of Immunopathology of Schistosomiasis (LIM-06), School of Medicine, University of São Paulo, 05403-000 São Paulo, SP, Brazil; ^2^University Center of Volta Redonda, 27240-560 Volta Redonda, RJ, Brazil; ^3^Department of Gastroenterology and Laboratory of Tropical Gastroenterology and Hepatology, School of Medicine, University of São Paulo, 05403-000 São Paulo, SP, Brazil; ^4^Department of Enteroparasites at the Parasitology and Mycology Service from the Adolfo Lutz Institute, 01246-902 São Paulo, SP, Brazil; ^5^Laboratory of Seroepidemiology and Immunobiology, Institute of Tropical Medicine, University of São Paulo, 05403-000 São Paulo, SP, Brazil; ^6^Laboratory of Medical Genomics, AC Camargo Cancer Center, 01509–010 São Paulo, SP, Brazil; ^7^Institute of Psychiatry (LIM-27), São Paulo Medical School, University of São Paulo, 01246-903 São Paulo, SP, Brazil; ^8^Section of Parasitology, Central Laboratory Division of Hospital das Clínicas, School of Medicine, University of São Paulo, 05403-000 São Paulo, SP, Brazil; ^9^Santa Casa Medical School, 01221-020 São Paulo, SP, Brazil; ^10^Institute of Tropical Medicine, University of São Paulo, 05403-000 São Paulo, SP, Brazil

## Abstract

Schistosomiasis constitutes a major public health problem, with an estimated 200 million people infected worldwide. Many areas of Brazil show low endemicity of schistosomiasis, and the current standard parasitological techniques are not sufficiently sensitive to detect the low-level helminth infections common in areas of low endemicity (ALEs). This study compared the Kato-Katz (KK); Hoffman, Pons, and Janer (HH); enzyme-linked immunosorbent assay- (ELISA-) IgG and ELISA-IgM; indirect immunofluorescence technique (IFT-IgM); and qPCR techniques for schistosomiasis detection in serum and fecal samples, using the circumoval precipitin test (COPT) as reference. An epidemiological survey was conducted in a randomized sample of residents from five neighborhoods of Barra Mansa, RJ, with 610 fecal and 612 serum samples. ELISA-IgM (21.4%) showed the highest positivity and HH and KK techniques were the least sensitive (0.8%). All techniques except qPCR-serum showed high accuracy (82–95.5%), differed significantly from COPT in positivity (*P* < 0.05), and showed poor agreement with COPT. Medium agreement was seen with ELISA-IgG (Kappa = 0.377) and IFA (Kappa = 0.347). Parasitological techniques showed much lower positivity rates than those by other techniques. We suggest the possibility of using a combination of laboratory tools for the diagnosis of schistosomiasis in ALEs.

## 1. Introduction

Schistosomiasis is a major public health problem, with 200 million people infected worldwide and 700 million people residing in areas of infection risk [1, 2].

In Brazil, schistosomiasis has been reported to occur in 19 states, and it is estimated that approximately 6 million people are infected and 25 million are at risk of contracting the disease. The national positivity rate is 6.94%, ranging from 0.04% in Piauí State to 11.88% in Pernambuco State. In Rio de Janeiro State, the positivity rate is 1.56% [3].

**Figure 1:**
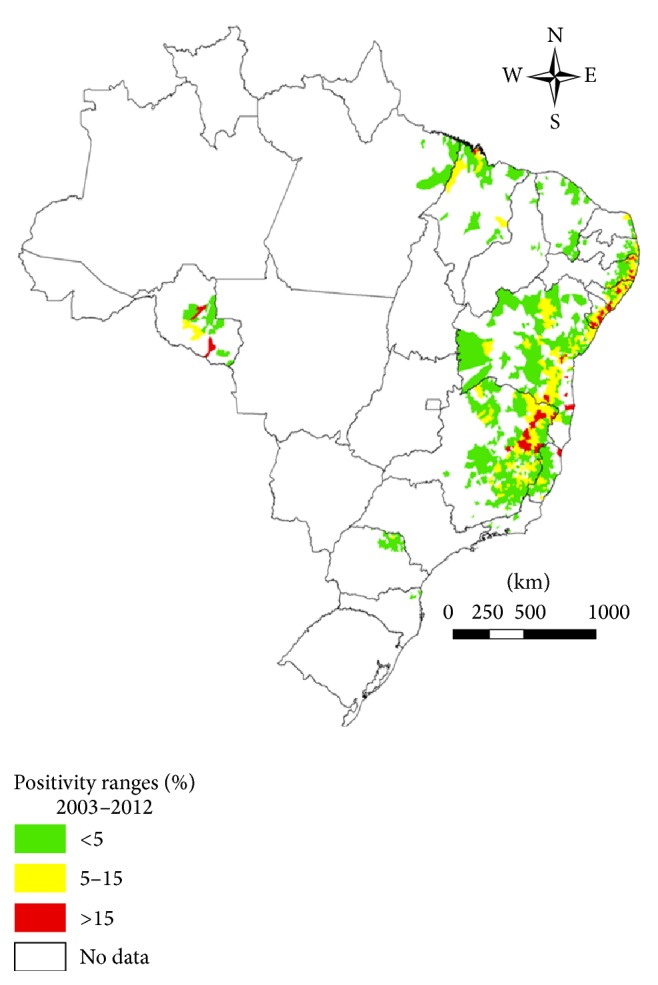
Distribution of positivity ranges for schistosomiasis based on the record of cases on investigated cities, Brazil, 2012. Source: SISPCE-SVS/MS.

Brazil has areas of different prevalence rates varying from state to state, as shown in Figure 1 [3].

Of the various known species of Schistosoma,* S. mansoni* has the widest global distribution and is the only species that causes schistosomiasis in Brazil [4].

Although the serious forms of schistosomiasis have become less prevalent, thanks mainly to the implementation of mass chemotherapy, the geographic expansion of schistosomiasis continues apace with the expansion of agricultural zones and irrigated areas [5].

The classification of the individual infection intensity criteria for* S. mansoni *is estimated by the quantity of eggs observed in parasitological examination of feces, using the Kato-Katz (KK) technique [2]. According to WHO, 2002 [6], the infection classification is high parasitic load (≥400 epg), medium parasite load (100–399 epg), and low parasite load (<100 epg) and areas with high, medium, or low endemicity show prevalence of ≥50%, ≥10% <50, or <10%, respectively. In ALEs, approximately 75% of infected individuals are asymptomatic, show few symptoms, and have low parasite load, which hinders diagnosis [6].

The state of Rio de Janeiro presents the lowest number of confirmed cases and deaths due to schistosomiasis in the southeast region of Brazil [3, 7]. The city of Barra Mansa is defined as microregion 2 of the Vale do Médio Paraíba, Rio de Janeiro State [8]. The city of Barra Mansa is located in the southern part of the state of Rio de Janeiro.

Barra Mansa is one of the foci of* S. mansoni* infection in the state of Rio de Janeiro [8]. The average prevalence was estimated to be 1%, from 2001 to 2008, based on the cases reported by the Notifiable Diseases Information System (SINAN) from 2001 to 2008 [9].

The endemic foci lie within the urban perimeter. The neighborhood of Siderlândia shows the highest prevalence, followed by the neighborhoods of Santa Clara, São Luiz, Cantagalo, and Nova Esperança. Isolated cases of infection by* S. mansoni* have been reported in further 30 neighborhoods [9].

Detection of* S. mansoni* eggs in feces has historically been used as the reference for diagnosing schistosomiasis, and* Schistosoma* species are identified by their characteristic morphology showing a lateral spicule. The parasitological methods are highly specific, inexpensive, and relatively simple to execute [2, 10–12]. The Kato-Katz (KK) technique is most commonly used for detecting* S. mansoni* eggs in epidemiological studies, allowing the quantification of eggs in fecal samples. The Hoffman technique (HH) is based on spontaneous sedimentation, and it is effective because embryonated* S. mansoni* eggs are heavy; however, it is not suitable for quantification of eggs in feces.

Although these parasitological methods are inexpensive and simple to perform, they lack sensitivity, especially in ALEs [13–18]. The Secretariat of Health Vigilance in Brazil has proposed the elimination of this form of helminthiasis. Therefore, there is a need to define alternative laboratory diagnostic techniques for detection of* S. mansoni* in ALEs. Thus, the aim of this study was to compare the efficiency of existing parasitological, immunological, and molecular diagnostic methods in areas of low prevalence of* S. mansoni*, using the circumoval precipitin test (COPT) as a reference, due to its high sensitivity and specificity in ALEs [19, 20].

## 2. Materials and Methods

### 2.1. Study Design, Population, and Sample Size

*S. mansoni* is endemic in the city of Barra Mansa, Rio de Janeiro State, Brazil, with an estimated prevalence of 1% [9]. Data for 2001–2008 from the Notifiable Diseases Information System (SINAN) showed that the disease is most prevalent in the neighborhoods of Siderlândia, Santa Clara, São Luiz, Nova Esperança, and Cantagalo, which belong to the Barra Mansa River Basin, a tributary of the Paraíba do Sul River. These five neighborhoods, located on the outskirts of the city of Barra Mansa, were selected for this cross-sectional study. Samples of feces and serum were collected from April to December 2011.

The sample size was calculated assuming a prevalence of 1%, with an addition of 30% to compensate for losses. The estimated sample size required was 650 individuals residing in the above neighborhoods. Households were systematically selected (one in six), and individuals were randomly selected by a draw among those who agreed to participate in the study. Subjects who were older than 5 years of age and had not been treated for* S. mansoni* in the last year were eligible for inclusion.

### 2.2. Statistical Analysis

Statistical analysis was performed using SPSS (Statistical Package for the Social Sciences) for Windows, version 15.0 (SPSS Inc., Chicago, IL, USA) and Microsoft Excel 2003. Significance levels were fixed by accepting a Type 1 error of 5% (*α* = 0.05). Population characteristics were described using absolute and relative frequencies and calculation of mean ages and standard deviations. All participants were evaluated by each diagnostic technique.

Each* S. mansoni* infection measurement technique was compared and marginal associations were verified using McNemar's test. Pairwise concordance between results was assessed using Cohen's kappa index and 95% confidence intervals. The Kappa index values range interpretation was as follows: poor agreement (<0.20); low agreement (0.20 to 0.40); moderate agreement (0.41 to 0.60); good agreement (0.61 to 0.80); and very good agreement (0.81 to 1.00) [21].

Associations between* S. mansoni* infection and age range, sex, neighborhood, river water use, and history of schistosomiasis were assessed for each technique using the Chi-square test, Fisher's exact, or likelihood ratio tests.

We compared the accuracy (sensitivity, specificity, likelihood ratio, and predictive values) of serological techniques to those of parasitological techniques. We also compared results among techniques to determine which were most effective in diagnosing* S. mansoni* in areas with a similar epidemiological profile to the target area of this study.

### 2.3. Ethical Aspects

In accordance with the rules governing human subject research, informed consent was obtained to meet the recommendations of Resolution n° 466 from December 12, 2012, of the National Council of Health. This research project was approved by the Research Ethics Committee of the Department of Infectious and Parasitic Diseases of the Faculty of Medicine of the University of São Paulo and the Research Ethics Committee of the Hospital das Clínicas (CAPPesq) of the Faculty of Medicine of the University of São Paulo (Approval number 0405/09).

The experimental research procedures met Laws 6.638/79 and 9605/98, Decree 24.645/34, the Ethical Principles of Animal Experimentation, the Principles for Research Involving Animals [22], and other directives governing animal research. The study began after approval of research project number CEP-IMT 2011/096 by the Animal Ethics Research Committee of the Institute of Tropical Medicine of São Paulo, University of São Paulo, Brazil.

### 2.4. Methods of Diagnostic Investigation

This was an interdisciplinary study, and the research laboratories developed their activities independently, following the conditions and operationalization timelines that respected the routine of each institute involved. Samples from all individuals in the study were subjected to the diagnostic techniques described and the reference diagnostic technique (COPT) [23]. The professionals who standardized and interpreted the COPT did not know the results for the diagnostic techniques, thus, reducing any interpretation bias.

The participating laboratories and the diagnostic techniques they developed were as follows: Municipal Secretary of Health of Barra Mansa/RJ (feces and serum collections); CentroLab Laboratory, Volta Redonda/RJ (preparing sample preanalysis); Institute Adolfo Lutz (COPT and ITF-IgM); Institute of Tropical Medicine/USP (ELISA); Laboratory of Gastroenterology and Tropical Hepatology at the Department of Gastroenterology/FMUSP (molecular biology), Research Center at A.C. Camargo Hospital (molecular biology), René Rachou Research Center/FIOCRUZ, Belo Horizonte/MG (molecular biology); Parasitological Section of the Central Laboratory, Division, HCFMU/SP (KK and HH).

The standardization and application of the diagnostic techniques were developed from December 2010 to December 2012, when the statistical analyses began.

### 2.5. Methods for Laboratory Diagnosis

The Family Health Program and health agents of the Municipal Schistosomiasis Control Program (PCE) collected 610 randomized fecal samples and 612 serum samples. Of these, 572 samples were paired, from inhabitants of 5 peripheral neighborhoods of Barra Mansa, Rio de Janeiro. The serum samples were aliquoted and stored at −20°C and then transported to São Paulo in thermal boxes containing dry ice and stored at −20°C in the lab until further testing.

### 2.6. Parasitological Methods

Stool samples were prepared following the KK (Helm Test, Bio-Manguinhos, Fiocruz, Rio de Janeiro, RJ, Brazil) and HH techniques and stored at 4°C until shipment. For the KK technique, two slides were prepared and stored in boxes fixed with a rigid polypropylene cover lined with cork (Prolab-Prolab, São Paulo, SP, Brazil). For the HH technique, samples were preserved in 10% formalin (Indalabor Indaiá Laboratório Farmacêutico Ltda., Dores do Indaiá, Minas Gerais, MG, Brazil), and two slides were prepared for each participant.

### 2.7. Laboratory Maintenance of the* S. mansoni* Experimental Cycle

The* S. mansoni* cycle was maintained through periodic infection of hamsters (*Mesocricetus auratus*) and* Biomphalaria glabrata* mollusks (strain BH). Each week, five animals were subcutaneously infected with 200–300 cercariae and sacrificed after 49 to 56 days to collect adult worms and parasite eggs.* S. mansoni* eggs were collected from liver granulomas. After washing with physiological solution for complete removal of blood, the worms and eggs were counted and frozen.

### 2.8. Adult Worm Total Extract

Approximately 10,000* S. mansoni* adult worms of both sexes were thawed and resuspended at a concentration of approximately 1,000 worms/mL in 10 mM phosphate buffered saline (PBS), at pH 7.2. The protease inhibitor phenylmethylsulfonyl fluoride (PMSF, Sigma-Aldrich, St. Louis, MO, USA) was then added to produce a final concentration of 1 mM. The suspension was ground in a manual tissue homogenizer in an ice bath for 1 h. After thorough mixing, the suspension was subjected to two centrifugations at 10,000 ×g, at 4°C for 45 min. The supernatant was then removed, and the protein content was measured using the DC Protein Assay reagent (Detergent-Compatible Colorimetric Assay Kit, Bio-Rad, Hercules, CA, USA) employing a modified Lowry method [24, 25]. The extract was aliquoted and stored in a freezer at −80°C until further use [26]. This total extract was used for the sensitization of microplates to IgG (ELISA-IgG) antibodies [27, 28].

### 2.9. TCA-Soluble Fraction

A soluble fraction in trichloroacetic acid (TCA-soluble fraction) was prepared from the crude extract of adult worms of* S. mansoni*, according to a previously described methodology [27], with modifications. An equal volume of 10% TCA was added to the total adult worm extract. After mixing vigorously for three cycles of 1 min, using a vortexer (Vortex Genie-2, Scientific Industries Inc., Bohemia, NY, USA), the suspension was subjected to centrifugation at 10,000 ×g, at 4°C for 45 min. The supernatant (containing the TCA-soluble fraction) was removed and then dialyzed against PBS on a cellulose membrane (Sigma-Aldrich) that retains substances with a molecular weight of 12,000 kDa or greater, with continuous stirring overnight at 4°C. We subsequently determined the protein content of the antigen solution (TCA-soluble fraction), which was then aliquoted and stored in a freezer at −80°C until further use. The TCA-soluble fraction was used for the sensitization of microplates to IgM (ELISA-IgM) antibodies [26, 28].

### 2.10. Enzyme-Linked Immunosorbent Assays (ELISA)

For ELISA-IgM, polystyrene plates (Costar High Binding 3590, Corning, NY, USA) were sensitized with 1 *µ*g/well of the TCA-soluble fraction of the* S. mansoni* total extract diluted in 0.1 M carbonate/bicarbonate buffer (pH 9.6), incubating for 2 h at 37°C, followed by 18 h at 4°C in a humid chamber. After washing three times with PBS containing 0.05% Tween-20 (PBS-T), the plates were blocked for nonspecific sites with 200 *µ*L of 5% skim milk in PBS-T (PBS-TM 5%), incubating for 2 h at 37°C in a humid chamber. The plates were then washed again with PBS-T. Serum samples were diluted (1 : 100) in PBS-T with 2% skim milk (PBS-TM 2%) and tested in duplicate (50 *µ*L/well). After incubation, for 30 min at 37°C in a humid chamber, the plates were washed three times with PBS-T, and 50 *µ*L of a *μ*-chain-specific anti-human IgM peroxidase conjugate (Sigma-Aldrich) diluted 1 : 5000 in PBS-TM 2% was added. After 30 min of incubation in a humid chamber, the plates were washed with PBS-T, and 100 *µ*L of a chromogenic mixture consisting of tetramethylbenzidine (TMB) and H_2_O_2_ was added. After 10 min of incubation in the dark, the reaction was quenched with 25 *µ*L of 2 N H_2_SO_4_. The absorbance at 450 nm was measured using an ELISA plate reader (Titertek Multiskan MCC/340, Lab Systems, Helsinki, Finland).

The reaction conditions described above for performing ELISA-IgM testing were also used for ELISA-IgG testing, except that polystyrene plates (Nunc PolySorp, Roskilde, Denmark), a 500 ng/well total antigen extract of* S. mansoni*, and an Fc-specific anti-human IgG peroxidase conjugate (Sigma-Aldrich) diluted 1 : 20,000 were used.

To determine the threshold of reactivity (cut-off) for ELISA-IgG and ELISA-IgM, receiver operating characteristic (ROC) curves were constructed. We used 13 and 29 samples from patients who tested positive and negative, respectively, for* S. mansoni* in the parasitological examination and IFT, and 13 samples from patients who tested positive for other helminth parasites in the parasitological examination and negative for* S. mansoni* in IFT.

**Figure 2:**
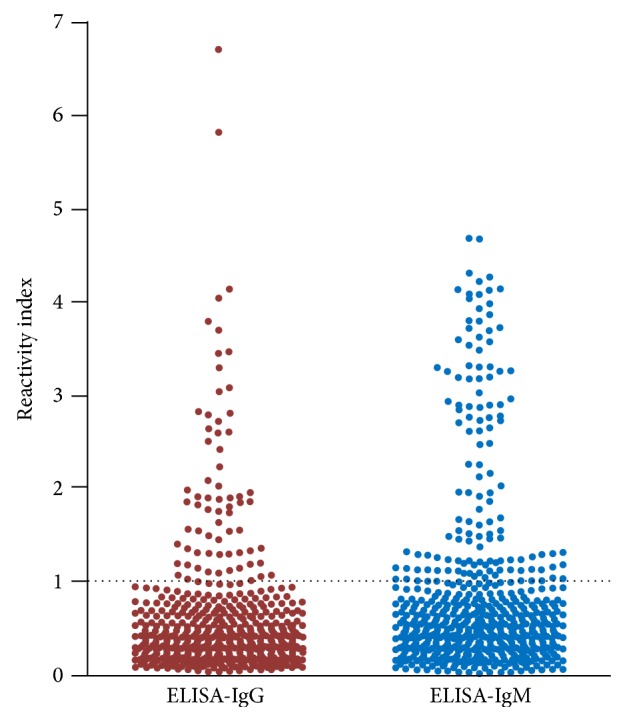
Reactivity index of 612 sera obtained by ELISA-IgG and IgM-ELISA technique of the individuals in the city of Barra Mansa, RJ, Brazil, 2011.

The reactivity index (RI) of the samples was calculated using the equation IR = sample absorbance/cut-off. Serum samples with IR ≥ 1.00 were considered reactive, Figure 2.

### 2.11. Detection of IgM Antibodies against Antigens of the* S. mansoni* Digestive Tract

IFT-IgM was used to detect antiantigen polysaccharide IgM antibodies in the digestive tract of adult* S. mansoni* worms on paraffin sections, according to the technique described by Silva et al. [29] and Nash, 1974 [30] and 1978 [31].

### 2.12. Obtaining Adult Worms

The infected hamsters were anesthetized with an intramuscular injection of 100 mg/kg of ketamine and xylazine and sacrificed, following the animal sacrifice guidelines of our institution. After sectioning the portal vein, 50 mL of 0.85% saline solution with ethylenediaminetetraacetic acid (EDTA) was infused in the left ventricle using a 20 mL syringe, through successive infusions, by not having an infusion pump for maintaining a continuous flow. Adult worms were obtained after perfusion of the portal system, removed from the abdominal cavity, and numbered and stored at −20°C. Adult male worms were separated for “particulate” antigen processing in paraffin sections for IFT-IgM analysis.

### 2.13. Slide Preparation for IFT-IgM

Approximately 60 adult male worms were placed on an end-jointed 200 *μ*L mesh screen (Tecmolin, PA-6-212/XX, São Paulo, SP, Brazil), immersed in Rossman's solution fixative for 2 h at room temperature and then immersed in 90% ethanol three times for 2 h. The samples were then immersed in absolute alcohol for 15 h and incubated in methyl benzoate for 4 h, xylol at 60°C for 15 min, 50% xylene/paraplast for 15 min, and 100% paraplast (Monjet Scient.) at 60°C for 30 min. The material was embedded in an L-shaped aluminum frame, placed at room temperature for 12 h, and subsequently stored at the same temperature for processing of histological sections. Serial sections of 5 *µ*m thickness were cut using a microtome, at ten sections per slide.

The slides containing paraffin sections were subjected to dewaxing and rehydration, using successive baths of xylene and ethanol at different concentrations, with a final bath in PBS, pH = 7.2. The slides were stored at room temperature until use.

### 2.14. IFT-IgM

Serum samples were diluted 1 : 10 in PBS solution (pH 7.2) and deposited on the paraffined sections of adult worms. After incubation at 37°C for 50 min in a humid chamber, slides were washed in 0,01 M PBS (pH 7.2) baths for 10 min. Anti-human IgM fluorescent conjugate (goat anti-human IgG *γ*-chain-specific fluorescein isothiocyanate antibody) was added (Sigma-Aldrich; St. Louis, MO, USA) in accordance with its optimal use titer (1/320) in PBS, pH = 7.2 containing 1% Evans blue solution (Bio-Rad Laboratories, Washington, DC, USA). After further incubation and washes, slides were dried and mounted with glycerol and coverslips. Positive standard serum was used for 1/10, 1/40, and 1/160 dilutions. Negative standard serum was used for 1/10 dilutions.

The IFT-IgM reading was performed using an Olympus BX-FLA fluorescence microscope (Olympus Corporation, Tokyo, Japan) equipped with an epi-illumination system, with 100x and/or 200x magnification.

**Figure 3:**
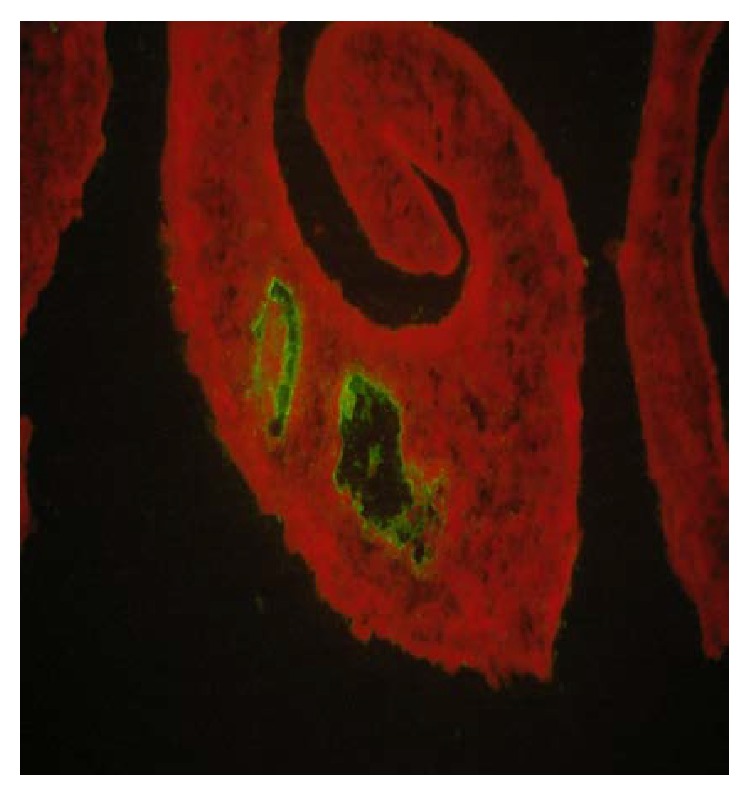
Reactivity of the inner lining of the digestive tube: positive human serum IgM antibodies.

A sample was considered positive when fluorescence was present only in structures related to the parasite digestive tract (Figure 3). Fluorescence detected in membranes or parasite parenchyma was considered nonspecific. The results were expressed as reactive (presence of fluorescence in the digestive tract) and nonreactive (absence of fluorescence) sera. The classification of the sera was based on the fluorescence intensity in the internal portion of the worms digestive tract (only male worms were used for embedment), according to a targeted fluorescence intensity scale. The attribution of 1+ to 4+ was obtained comparing the results with a photographic standard built with serum: nonreactive (0), low (1+), medium (2+), strong (3+), and very strong (4+).

### 2.15. Antibody Detection of* S. mansoni* Egg Antigens

Circumoval precipitin test (COPT) was used to detect antibody reactions against excretion and secretion products of* S. mansoni* eggs, using previously described techniques [32].

### 2.16. Isolation and Purification of* S. mansoni* Eggs

*S. mansoni* eggs were isolated and purified as described by Dresden and Payne [33] and Pinto et al. [34] with some modifications.

Livers from three infected hamsters were cut into small pieces (3 mm) and incubated in a water bath at 37°C for 20 min, in a solution containing 0.004% pepsin and 0.7% hydrochloric acid. After incubation, the peptic solution was discarded and the tissue fragments were added to 150 mL of 0.9% ice-cold saline solution containing 50 *µ*L of Triton X-100. The material was homogenized using several drive pulses in a domestic blender (Walita Philips, Amsterdam, Netherlands) until the fragments were completely ruptured. The material was then filtered in fourfold gauze and screen processed under negative pressure through a series of metallic sieves with mesh numbers 100 (0.150 mm), 200 (0.075 mm), and 400 (0.038 mm) (Granutest, Telastem, Peneiras Para Análise Ltda., São Paulo, SP, Brazil).

Eggs retained on the last sieve were removed by successive washing with ice-cold 0.9% saline solution and concentrated to 1 mL volume by centrifugation at 154 ×g for 15 sec. From this concentrated suspension, a 10 *µ*L aliquot was placed between the blade and the coverslip and evaluated under a microscope (Olympus Corporation, Tokyo, Japan) at 100x magnification. The number of eggs under the entire area of the coverslip was counted. Suspensions exceeding 20% cell debris in relation to the number of eggs found were reprocessed through the 200 and 400 mesh sieves. For COPT, the egg suspensions were adjusted to contain 300 viable eggs per 10 *µ*L of 0.9% saline solution and stored at 4°C for 4 h until reaction preparation.

### 2.17. Circumoval Precipitin Test

**Figure 4:**
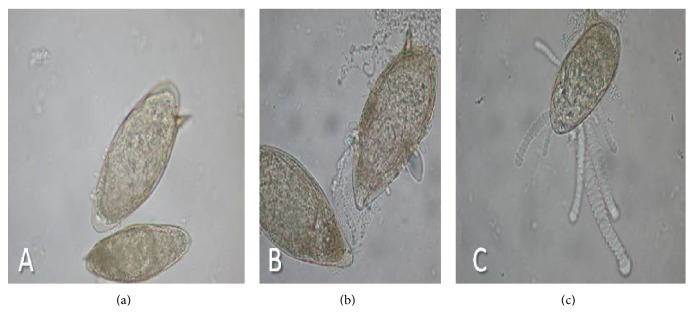
Intensity patterns of RPO: (a) RPO pattern 1; (b) RPO pattern 2; (c) RPO pattern 3.

Aliquots (10 *µ*L) of each purified egg suspension, adjusted to contain 300 viable eggs in 0.9% saline solution, and 50 *µ*L of each serum sample, were added to 2 mL Eppendorf tubes (Eppendorf do Brasil Ltda., São Paulo, SP, Brazil) and incubated at 37°C for 48 h. Subsequently, a 30 *µ*L aliquot of each mixture (serum + viable egg suspension) was placed on a slide and covered with a coverslip (22 × 22 mm). Assessment was performed on a binocular Olympus-CX41 microscope (Olympus Corporation, Tokyo, Japan), with a 10x or 40x objective lens. The number of reactive eggs per 100 viable eggs and the periovular precipitate morphology were used for assessment.* S. mansoni* eggs were considered reactive if they contained globular precipitates of variable size and form, or if they appeared as small or long septated chains, similar to* Taenia* segments (Figure 4). Sera were considered positive if at least 9% of the mature eggs were reactive in the presence of different precipitates [35].

### 2.18. TaqMan Real-Time PCR Assays

Preanalysis preparation of feces samples for qPCR. Fecal samples, without conservatives, were passed through a Nylon Baltex PA-7-200/XX filter (Tecmolin, São Paulo, SP, Brazil) to remove larger impurities. Using a marked stainless steel measuring plate, approximately 500 mg of feces from each sample was aliquoted and stored in a freezer at −20°C.

### 2.19. DNA Extraction from Serum

DNA was extracted from hamster serum samples and the 0.9% saline solution containing 200 eggs/mL, using the guanidine isothiocyanate-phenol-chloroform (GT) method [36, 37]. DNA was stored at −20°C after extraction.

### 2.20. DNA from Fecal Samples

DNA extraction from hamster feces was performed in two phases: in the first phase, after resuspending approximately 500 mg of stool in 1 mL 0.1 M PBS, five glass beads were added. This mixture was homogenized for 5 min using a vortex mixer, followed by centrifugation for 8 min at 16.863 ×g at 4°C. An aliquot of 400 *μ*L of the supernatant was mixed with 100 *µ*L of Rapid One-Step Extraction (ROSE) solution: 10 mM Tris-hydroxymethyl amino methane HCl, pH 8; 300 mM EDTA, pH 8.0; 1% sodium lauroyl sarcosinate (Sarkosyl); and 1% polyvinylpolypyrrolidone (PVPP) [38]. Then, 30 *μ*L of Proteinase K (Life Technologies, Carlsbad, CA, USA) was added and homogenized using a vortex mixer. The sample was incubated for 120 min at 65°C. In the second phase, the DNA present in this solution was extracted and stored as described for serum samples.

### 2.21. Purification of DNA Extracted from Fecal and Serum Samples

DNA extracted from serum and feces, at distinct times after infection, was purified using the InstaGene Matrix in accordance with the manufacturer's instructions. Once purified, extracted DNA was stored at −20°C until use.

### 2.22. Amplification of DNA from Serum and Fecal Samples

*Primers and Probes.* A set of primers and probes complementary to a 121 bp tandem repeat sequence from* S. mansoni* strain SM 1-7 (GenBank accession number M61098), described by Hamburger et al. [39], were used for amplification and detection of* S. mansoni* DNA. Primer sequences were as follows: forward F2: 5′-CCG ACC AAC CGT TCT ATG A-3′; reverse R2: 5′-CAC GC TCT CGC AAA TAA TCT AAA-3′; probe PO2: 5′-6[FAM] TCG TTG TAT CTC CGA AAC CAC TGG ACG [(BHQ1])-3′; all probes were synthesized by Sigma Life Sciences (Woodlands, TX, USA).

All samples were evaluated using TaqMan Reagents Exogenous Internal Positive Control (IPC) (Life Technologies), in accordance with the manufacturer's instructions to test for the presence of Taq DNA polymerase inhibitors.

For the positive control of the qPCR-feces, in all assays, we used DNA obtained in the extraction of 200 eggs/mL of saline 0.9%.

### 2.23. TaqMan Real-Time PCR Conditions for Serum and Fecal Samples

TaqMan Real-Time PCR was performed in a final volume of 20 *μ*L containing 10 *μ*L TaqMan Universal PCR Master Mix 2X, 20 pmol of primers F2 and R2, 5 pmol of the PO2 probe, and 2 *μ*L of purified DNA. For each sample, another reaction was performed in parallel using the TaqMan Reagents Exogenous Internal Positive Control (IPC) in a final volume of 21 *μ*L, containing 10 *μ*L of TaqMan Universal PCR Master Mix 2X, 5 *μ*L 10X Exogenous IPC mix, 1 *μ*L 50X Exo IPC, and 5 *μ*L of purified DNA samples. For each batch of reactions, two additional controls were used: a no amplification control (NAC) and a no template control target (NCT). PCR was performed in an Applied Biosystems 7300 Real-Time PCR System (Life Technologies) using the following cycling conditions: 50°C for 2 min; 95°C for 10 min; and 40 cycles at 95°C for 15 sec and 60°C for 1 min.

To detect* S. mansoni*, the Applied Biosystems 7300 Real-Time PCR System standard cycles were used: 50°C for 2 min; 95°C for 10 min; 40 cycles at 95°C for 15 sec, and 60°C for 1 min.

For the positive control of the qPCR-serum, in all assays, we used DNA obtained in the extraction of  200 eggs/mL of saline 0.9%.

Furthermore, the possibility of contamination was minimized by performing DNA extraction and amplification in separate rooms, by performing all experiments inside a laminar flow cabinet, by frequent use of ultraviolet (UV) (Bio II A-Telstar, Life Science, Vancouver, Canada) irradiation, and by using only disposable, sterile laboratory equipment and pipette tips with filters.

### 2.24. Criteria Used to Evaluate the Results of qPCR Using Stool and Serum Samples

The following criteria were used to evaluate the qPCR:

 
*Positive qPCR*: duplicates of the sample were amplified by qPCR.
 
*Undetermined qPCR*: one aliquot of the sample was amplified by qPCR.
 
*qPCR IPC*: exogenous control for the testing sample was not amplified (negative) by qPCR.

The undetermined qPCR and qPCR IPC samples were tested in triplicate. Duplicate amplifications of the samples obtained by qPCR were included in the positive results [38].

## 3. Results

The characteristics of the sample population (650 freely participating individuals) residing in the five suburbs of the city of Barra Mansa (RJ), used to evaluate the techniques ELISA-IgG, ELISA-IgM, ITF-IgM, COPT, qPCR-serum, qPCR-feces, KK, and HH, are shown in Table 1.

Analysis of Table 1 reveals that the majority of the individuals were female with an average age of 39.7. The greatest number of participants resided in the neighborhood of Sinderlândia (42.9%) and approximately 4% of the participants reported a history of schistosomiasis.

The positivity for infection by* S. mansoni* according to each diagnostic technique used in this study is shown in Table 2.

The technique that presented most evidence of infection by* S. mansoni* was the ELISA-IgM (21.4%), whereas the parasitological techniques (KK and HH) showed the lowest rates of infection (0.8%).

In the KK technique, the quantity of eggs detected per gram of feces varied from 0 to 456 eggs. One of the five positive cases was diagnosed only with the HH technique.

The prevalence of infection with* S. mansoni* and various enteroparasites diagnosed by the KK and HH techniques in individuals from the city of Barra Mansa/RJ, 2011, is shown in Table 3.

Of the individuals tested, 27.9% were found to be infected with at least one parasite.

The results of the KK technique showed low positivity for any diagnosed parasitosis, and the positivity rate for infection by* S. mansoni* was 0.7% (*n* = 4).

The ELISA-IgG reactivity index showed an average of 2.12, with an SD of 1.10 (median = 1.85; minimum = 1.01; maximum = 6.72; *n* = 71). The ELISA-IgM reactivity index showed an average of 2.24, with an SD of 1.09 (median = 1.91; minimum = 1.02; maximum = 4.67; *n* = 131). The IFT-IgM positivity showed an average of 1.56 crosses, with an SD of 0.69 (median = 1; minimum = 1; maximum = 3; *n* = 97). COPT reactivity was observed in an average of 21% of viable eggs, with an SD of 9% (median 21% viable eggs; minimum = 9% viable eggs; maximum 38% viable eggs; *n* = 33).

Table 4 shows that the results of the qPCR technique that were positive (Figure 6) and undetermined occurred with a frequency of 9.8% and 8.9% and a median of 34.8 and 37.1, respectively.

In this study, we found that in 16.2% (*n* = 99) of the fecal samples amplification of the exogenous control (IPC) and* S. mansoni* DNA was unsuccessful.

In serum samples (Table 5), the Ct values of the qPCR positive (Figure 6) and undetermined samples were 1.5% (*n* = 9) and 5.1% (*n* = 31) and the median was 36.3 and 37.0, respectively.

Table 6 shows that the positivity rate of the COPT technique differed significantly from all other techniques; it showed a lower positivity rate than the ELISA-IgG, ELISA-IgM, IFT-IgM (*P* < 0.001), and qPCR-feces (*P* = 0.001) techniques and a higher positivity rate than the KK, HH, and qPCR-serum techniques (*P* < 0.001). The results of the COPT technique presented greater concordance with those from ELISA-IgG (Kappa = 0.377), IFT-IgM (Kappa = 0.347), and qPCR-feces (Kappa = 0.311) techniques.

When comparing COPT with the other diagnostic techniques, ELISA-IgM assay presented the highest sensitivity (Table 7).

## 4. Discussion

Diagnosis of* S. mansoni* is challenging, and determining the prevalence of infection is therefore difficult. Schistosomiasis is a chronic infection that may not progress to a severe form but that can trigger debilitating sequelae that are secondary to parasitism. This is particularly true in children, who may experience delayed growth and develop anemia and chronic malnutrition [2]. Moreover, asymptomatic carriers can potentially transmit schistosomiasis in areas that lack adequate sanitation [40].

Because of the present state of endemism of schistosomiasis in Brazil, the current proposal is the interruption (elimination) of its transmission. Thus, there is a need to develop new techniques that are more sensitive and specific, to permit early diagnosis and treatment of infected individuals, necessary for the control of this helminthiasis [3].

In this study, we compared the performance of the KK and HH techniques, the ELISA-IgG, ELISA-IgM, and the ITF-IgM techniques, as well as qPCR of stool and serum samples, using the COPT technique as a reference [19, 20, 41].

The positivity rate for schistosomiasis in our study population, as determined by immunological techniques, ranged from 21.4% (131/612) for ELISA-IgM to 15.8% (97/612) for ITF-IgM and 11.6% (71/612) for ELISA-IgG. These rates are above those for parasitological techniques to diagnose* S. mansoni* infection. The low sensitivity of the parasitological method in ALEs had previously been described [13–18, 28, 42, 43].

The high positivity rates observed in this study for the ELISA-IgG, ELISA-IgM, and ITF-IgM techniques could have been caused by inclusion of individuals previously infected by* S. mansoni*, but properly treated and cured, or inclusion of those that were exposed to very small loads of cercariae or unisexual infections [16, 44, 45]. The possibility of cross-reactivity with cercariae antigens from parasites that infect other animal species and cross-reactivity with other parasites must also be considered [28, 46–48].

The positivity rate of the COPT technique also differed significantly from the positivity rates of the parasitological techniques (*P* < 0.001). The level of concordance (*k* = 0.224) for these techniques was higher than that observed for the other immunodiagnostic techniques (ELISA-IgG, ELISA-IgM, and ITF-IgM).

This higher positivity rate observed for the COPT could be due to the fact that oviposition occurs with egg deposition in the deeper layers of the intestine, even when eggs are absent from the feces, mainly in chronic infections in patients over 40 years of age and in schistosomiasis infections with low parasite load [49, 50].

The COPT positivity was lower than that for the ELISA-IgG and ELISA-IgM and ITF-IgM tests, probably as a result of the different nature of the antigens. The COPT detects antibodies against excretion products and secretions of miracidia or antibodies against antigens present in the fluid surrounding* S. mansoni* eggs, whereas the ELISA-IgG, ELISA-IgM, and ITF-IgM techniques detect antibodies against antigens in the internal coating of the digestive tube of adult worms [32, 50].

The appearance of the antibodies detected by the COPT is precocious and coincides with the beginning of the elimination of the eggs in the feces [49, 51, 52]. Thus, the antibodies detected by the COPT are only produced when worms of both sexes cause the infection; the ELISA-IgG, ELISA-IgM, and ITF-IgM techniques can also detect unisexual infections [44, 52, 53].

To the best of our knowledge, this is the first time that qPCR-feces and qPCR-serum were used in a population study in low endemicity area for* S. mansoni* infection.

The qPCR-feces technique exhibited 12-fold higher positivity than the KK and HH techniques, at 9.8% (*n* = 60) compared to 0.8% (*n* = 5) for the parasitological techniques. The qPCR-feces positivity rate was only higher than the rates for the COPT (5.4%) and the qPCR-serum (1.5%) techniques; its results differed significantly from all techniques (*P* < 0.05) except the ELISA-IgG technique.

The positivity rate of the qPCR-serum technique was higher than those of the parasitological techniques but lower than those of the other techniques, including the qPCR-feces technique. A study by Pontes et al. [23] also indicated that the qPCR-serum technique has lesser sensitivity than the conventional PCR-feces technique for detecting* S. mansoni* infection. However, Wichmann et al. [54] and Zhou et al. [55] correlated the positivity in the acute phase of schistosomiasis infection with inflammatory alterations. The acute phase would cause increased circulation of schistosomiasis degradation products, both adult worms and eggs, thus increasing the quantity of circulating DNA. This phase would usually continue until the eighth week after infection, and these data would explain the higher rate of amplification using the qPCR-serum technique.

The median of the Ct values of the logarithmic curves for the qPCR-feces and serum techniques was 34.8 and 36.3, respectively, indicating low amplification of the DNA template, found in egg fragments (0.02 eggs at a dilution of 1/10,000), based on the standardization of the technique. The low parasite load in* S. mansoni* infections observed in ALEs could explain this result [56].

Wichmann et al. [57] reported a higher positivity rate for the qPCR-serum technique in patients with acute schistosomiasis, using the presence of* S. mansoni* DNA in duplicate samples as a criterion for positivity, with a limit of Ct < 45.

In spite of the presence of factors that inhibit the qPCR-feces technique and the low parasite load in the sampled population, the qPCR-feces technique showed a 12-fold higher positivity rate (60/610) than the KK and HH (5/610) techniques; the qPCR-serum technique presented a positivity (9/612) of, approximately, twofold higher than those of the parasitological techniques.

According to Cnops et al. [58], all PCR-based techniques for genomic DNA of* S. mansoni* were able to detect the DNA from all phases of the life cycle of this parasite: eggs containing miracidia, cercariae, schistosomulae, and adult worms. In summary, a positive PCR assay indicates the presence of the parasite but does not provide information regarding its life cycle phase or its viability, including the presence of mature, male, and female worms capable of egg deposition. This is likely to explain why we have not found a positive correlation between the Ct value and the number of eggs.

The technique developed by Kato and Miura [10], modified by Katz et al. [59], became the international reference technique for diagnosing schistosomiasis infection [60]. However, a decline in the sensitivity of this technique was observed in individuals with low parasite load infections residing in ALEs, following specific treatment, which compromises the evaluation of new techniques for diagnosing infection by* S. mansoni*, when it is used as a reference [61, 62].

Various studies have been performed to identify alternative methods of diagnosis applicable on a large scale that could be used to aid the programs for epidemiological vigilance in areas where, despite control actions, new cases of confirmed autochthony still occur [63].

Immunodiagnostic techniques are still used in schistosomiasis control programs in countries such as China and Venezuela [43, 50, 64, 65]. In Brazil, various techniques have been tested in regions considered to have low endemicity, such as in the states of São Paulo and Rio de Janeiro [28, 40, 48, 66–68].

The COPT is considered by some researchers to be the gold-standard technique for diagnosing schistosomiasis in ALEs [69, 70].

Taking into account the possibility of employing parasitological and immunological techniques for diagnosing schistosomiasis infection in ALEs, Alarcón de Noya et al. [50] proposed three laboratory criteria for case definition in these areas:

- individuals eliminating* S. mansoni* eggs in feces. In this group, COPT and serological positivity reactions are common;
- individuals without* S. mansoni* eggs in their feces, with positive COPT who have not received specific treatment in the previous 12 months. Usually, this group shows positivity for one and/or two serological techniques;
- individuals without* S. mansoni* eggs in their feces with negative COPT who have not received specific treatment in the previous 12 months but who have shown positivity for two serological techniques.

Use of criteria (I) and (II) presented above would yield 34 positive cases in our study population, and the prevalence would be, approximately, 5.6% (34/612) greater than the prevalence detected by the parasitological techniques 0.8% (5/612) prevalence detected by the parasitological techniques used in the program for schistosomiasis vigilance.

As we verified in this study, various techniques could be used for diagnosis of schistosomiasis; however all present problems of sensitivity or specificity. Thus, we could not identify in this study the ideal technique that combines high rates of sensitivity and specificity and low cost and easy applicability, even in the field, probably due to the low prevalence of infection (approximately 1%) in the area studied. All assays employed showed low sensitivity and specificity when applied to the study population. Low parasite load, very recent infections, and the low number of positive parasitological exams may explain this.

One limitation of this study is the collection of a single fecal sample per individual as recommended by the guidelines of the National Program for Schistosomiasis, since the sensitivity of the KK test could have been improved by testing samples collected on two or three successive days to compensate for daily variation in egg production in an infected individual.

The present study indicates that the use of combined tools can improve the diagnosis of low parasite load* S. mansoni* infections, in both clinical and epidemiological studies. The benefits of parasitological techniques for the control of* S. mansoni* are widely recognized, particularly the KK technique. We consider the presence of ALEs to be an indicator of the positive outcome of schistosomiasis control programs worldwide. However, in order to eliminate the risk of schistosomiasis in populations of low endemicity areas, the pursuit of novel techniques must be considered. Potentially effective methods may include novel immunodiagnostic techniques, molecular biology tools, and parasitological techniques, such as saline gradient, and magnetic bead-based isolation of eggs [26, 37, 71–75]. The development of these tools and the subsequent elimination of schistosomiasis will be a great challenge that will require the combined efforts of governments, health professionals, and researchers.

To advance this agenda, we suggest a change to the third criterion proposed by Alarcón de Noya et al. [50], considering cases of schistosomiasis in which individuals present positive qPCR-serum and/or qPCR-feces results. We withdrew the criterion regarding positivity in two serological techniques and negative results in the parasitological and the COPT techniques (Table 8).

With regard to field activities, we propose serological and parasitological survey of ALEs. We recommend the use of the ELISA-IgM and ELISA-IgG techniques for an initial triage of the target population, due to their greater sensitivity, to the possibility of automation, and to their ability to provide quantitative results. We suggest the use of the KK and HH parasitological techniques for evaluating the intensity of* S. mansoni* infections and identifying the presence of other parasitic infections, respectively.

**Figure 5:**
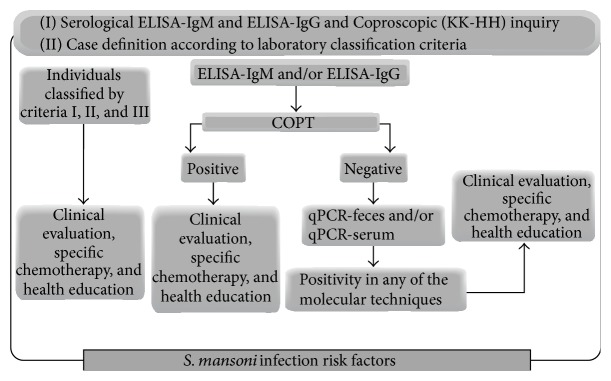
Proposal for epidemiological vigilance in ALEs.

**Figure 6:**
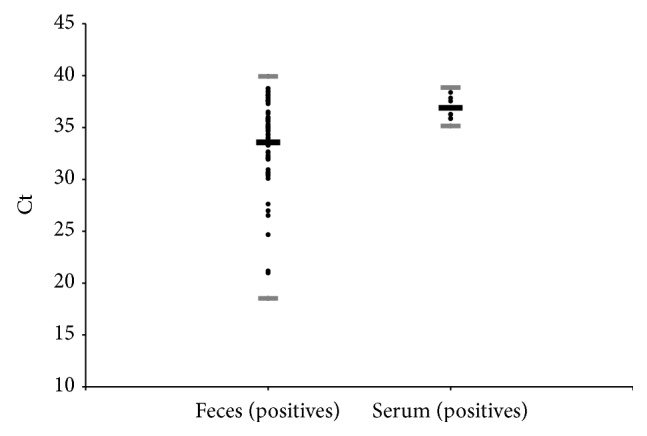
Description of the threshold cycle (Ct) for the results from the positive and qPCR in serum and feces samples from the individuals sampled from the city of Barra Mansa/RJ, 2011.

Following the triage, we recommend that actions be determined according to laboratory classification criteria. Thus, individuals classified under criteria (I), (II), and (III) would be referred for clinical evaluation, specific chemotherapy, and health education. For individuals with positive results for one and/or two serological techniques (ELISA-IgG or ELISA-IgM), we propose performing the COPT. For the patients who present a positive COPT, we recommend a clinical evaluation, treatment, and health education. If the result of the COPT is negative, we recommend performing qPCR-feces and qPCR-serum; if any of these molecular techniques yield a positive result, we suggest clinical evaluation, specific treatment, and health education (Figure 5).

We propose the combination of the ELISA-IgM and IgG techniques in the triage phase due to the possibility of automation, and to their higher levels of sensitivity, thus enabling the identification of a greater number of cases. In addition, the ELISA-IgM technique is more sensitive and the ELISA-IgG technique is more specific; hence, their combination will improve diagnosis.

According to our proposal, positivity of one or two immunological techniques indicates the need for confirmation by the COPT, which, despite being more difficult to perform, can identify active infections, or by the molecular reactions, which can identify the presence of the parasite at any stage of infection.

The possibility of the use of rapid tests with recombinant antigens [76] or with Circulating Anodic Antigen (CAA) in urine, feces, or serum [77–80] might be an interesting alternative for field works in places where health workers do not have the proper training and equipment to perform sophisticated serological assays.

However, continued use of the parasitological techniques will be important, in particular the HH technique, as it permits the evaluation of the parasite load in positive cases and allows observation of different levels of prevalence as well. Besides the evaluation and treatment, the infection risk factors must be taken into account.

We believe that maintaining this level of surveillance in ALEs will present a great challenge and will require evaluation of costs, interactions with other studies, and finally a broad discussion involving the various actors in the context of public health, research, and society.

In Brazil, recent epidemiological data on* S. mansoni* point to an increase in ALEs, and the current proposal aims to interrupt the transmission of this helminth (Ordinance number 2,556 of 28 October 2011). In this context, diagnosis of* S. mansoni* becomes a strategic hurdle we must overcome in order to reach the desired result indicators in the process of eliminating this parasitic infection. In addition, the development of new diagnostic laboratory techniques for detecting low parasite load infections represents an important strategy to overcome this need, in clinical contexts as well as in public health.

## Figures and Tables

**Table 1:** Sociodemographic characteristics of the study population, individuals from the city of Barra Mansa, RJ, 2011.

Characteristic	Frequency	%
Sex		
Female	385	59.2
Male	265	40.8
Age group (years)		
1 to 9	27	4.2
10 to 19	144	22.2
20 to 49	250	38.5
50 or over	229	35.2
Average age (SD)	39.7 (21.1)	
Literacy		
Yes	624	96.0
No	22	3.4
Not reported	4	0.6
Neighborhood		
Cantagalo	47	7.2
Nova Esperança	187	28.8
Santa Clara	35	5.4
São Luiz	102	15.7
Siderlândia	279	42.9
Water supply		
General network	551	84.8
Well or spring	79	12.2
Others	1	0.2
Not reported	19	2.9
Use of river water		
No	473	72.8
Washing clothes	15	2.3
Washing utensils	2	0.3
Baths	5	0.8
Swimming	5	0.8
Sand extraction	7	1.1
Not reported	143	22.0
Destination of feces and urine		
Sewer system	486	74.8
Septic tank	7	1.1
In the open	117	18.0
Not reported	40	6.2
Previous schistosomiasis		
Yes	25	3.8
No	519	79.8
Not reported	106	16.3

**Table 2:** Positivity for infection by *S. mansoni*, according to diagnostic technique, in samples collected from individuals in the city of Barra Mansa, RJ, 2011.

Technique	Positive/total	%
KK-HH	5/610	0.8
ELISA-IgG	71/612	11.6
ELISA-IgM	131/612	21.4
COPT	33/612	5.4
ITF-IgM	97/612	15.8
qPCR-feces	60/610	9.8
qPCR-serum	9/612	1.5

**Table 3:** Prevalence of schistosomiasis mansoni and other enteroparasites, as determined using the KK and HH techniques in individuals from the city of Barra Mansa, RJ, 2011.

Parasitosis HH/KK	Frequency	%
*Schistosoma mansoni *	5	0.8
*Endolimax nana *	106	17.4
*Entamoeba coli *	28	4.6
*Entamoeba hartmanni *	1	0.2
*Entamoeba histolytica/dispar *	5	0.8
*Blastocystis *spp.*/hominis *	66	10.8
*Giardia lamblia/intestinalis *	11	1.8
*Iodamoeba butschlii *	1	0.2
*Enterobius vermicularis *	6	1.0
*Strongyloides stercoralis *	9	1.5
*Ascaris lumbricoides *	4	0.7
*Trichuris trichiura *	3	0.5
*Taenia *sp.	2	0.3

**Table 4:** Results from the positive and undetermined qPCRs, with the threshold cycle (Ct) values, in fecal samples from the population of the city of Barra Mansa, RJ, 2011.

Technique	Cases/total	%	Ct values (LOG) qPCR				
			Average	SD	Median	Minimum	Maximum
qPCR positive	60/610	9.8	33.6	4.9	34.8	14.7	38.8
qPCR undetermined	54/610	8.9	32.3	8.5	37.1	11.5	40.0

**Table 5:** Description of the threshold cycle (Ct) for the results from the positive and undetermined qPCR in serum samples from the individuals sampled from the city of Barra Mansa, RJ, 2011.

Sample	Technique	Cases/total	%	Ct values (LOG) qPCR				
				Average	SD	Median	Minimum	Maximum
Serum	qPCR positive	9/612	1.5	36.9	1.3	36.3	38.8	35.2
	qPCR undetermined	31/612	5.1	35.5	4.4	37	39.7	20.3

**Table 6:** Concordance between the positive results obtained using the COPT technique and those obtained using the other techniques, in fecal and serum samples collected from the sample population in the city of Barra Mansa, RJ, 2011.

Techniques	COPT		Total				IC (95%)	
	Negative	Positive			*P* McNemar	Kappa		
	*n* (%)	*n* (%)	*n* (%)				Lower	Higher
KK-HH								
Negative	542 (94.8)	25 (4.4)	567 (99.1)		**<0.001**	0.224	0.038	0.409
Positive	1 (0.2)	4 (0.7)	5 (0.9)					
Total	543 (94.9)	29 (5.1)	572 (100.0)					
ELISA-IgG								
Negative	530 (86.6)	11 (1.8)	541 (88.4)		**<0.001**	0.377	0.255	0.500
Positive	49 (8.0)	22 (3.6)	71 (11.6)					
Total	579 (94.6)	33 (5.4)	612 (100.0)					
ELISA-IgM								
Negative	475 (77.6)	6 (1)	481 (78.6)		**<0.001**	0.266	0.178	0.354
Positive	104 (17.0)	27 (4.4)	131 (21.4)					
Total	579 (94.6)	33 (5.4)	612 (100.0)					
IFT-IgM								
Negative	508 (83.0)	7 (1.1)	515 (84.2)		**<0.001**	0.347	0.241	0.454
Positive	71 (11.6)	26 (4.2)	97 (15.8)					
Total	579 (94.6)	33 (5.4)	612 (100.0)					
qPCR-feces								
Negative	503 (87.9)	14 (2.4)	517 (90.4)		**0.001**	0.311	0.176	0.446
Positive	40 (7.0)	15 (2.6)	55 (9.6)					
Total	543 (94.9)	29 (5.1)	572 (100.0)					
qPCR-serum								
Negative	574 (93.8)	29 (4.7)	603 (98.5)		**<0.001**	0.171	0.013	0.330
Positive	5 (0.8)	4 (0.7)	9 (1.5)					
Total	579 (94.6)	33 (5.4)	612 (100.0)					

**Table 7:** Description of the sensitivity, specificity, the positive likelihood ratio, the negative likelihood ratio, the positive predictive value, and the negative predictive value of all diagnostic techniques compared to the COPT technique in individuals from the city of Barra Mansa, RJ, 2011.

Techniques	Parameters	Estimate	IC (95%)	
			Lower	Higher
KK-HH	Sensitivity (%)	13.8	3.9	31.7
	Specificity (%)	99.8	99.0	100.0
	Likelihood ratio (+)	74.9	8.6	649.0
	Likelihood ratio (−)	0.9	0.7	1.0
	Positive predictive value (PPV) (%)	80.0	28.4	99.5
	Negative predictive value (NPV) (%)	95.6	93.6	97.1
	Accuracy (%)	95.5	95.1	95.8

ELISA-IgG	Sensitivity (%)	66.7	48.2	82.0
	Specificity (%)	91.5	89.0	93.7
	Likelihood ratio (+)	7.9	5.5	11.3
	Likelihood ratio (−)	0.4	0.2	0.6
	Positive predictive value (PPV) (%)	31.0	20.5	43.1
	Negative predictive value (NPV) (%)	98.0	96.4	99.0
	Accuracy (%)	90.2	89.5	90.9

ELISA-IgM	Sensitivity (%)	81.8	64.5	93.0
	Specificity (%)	82.0	78.7	85.1
	Likelihood ratio (+)	4.6	3.6	5.8
	Likelihood ratio (−)	0.2	0.1	0.4
	Positive predictive value (PPV) (%)	20.6	14.0	28.6
	Negative predictive value (NPV) (%)	98.8	97.3	99.5
	Accuracy (%)	82.0	80.9	83.2

IFT-IgM	Sensitivity (%)	78.8	61.1	91.0
	Specificity (%)	87.7	84.8	90.3
	Likelihood ratio (+)	6.4	4.9	8.5
	Likelihood ratio (−)	0.2	0.1	0.5
	Positive predictive value (PPV) (%)	26.8	18.3	36.8
	Negative predictive value (NPV) (%)	98.6	97.2	99.5
	Accuracy (%)	87.3	86.4	88.1

qPCR-feces	Sensitivity (%)	51.7	32.5	70.6
	Specificity (%)	92.6	90.1	94.7
	Likelihood ratio (+)	7.0	4.4	11.1
	Likelihood ratio (−)	0.5	0.3	0.8
	Positive predictive value (PPV) (%)	27.3	16.1	41.0
	Negative predictive value (NPV) (%)	97.3	95.5	98.5
	Accuracy (%)	90.6	89.9	91.3

qPCR-serum	Sensitivity (%)	12.1	3.4	28.2
	Specificity (%)	99.1	98	99.7
	Likelihood ratio (+)	14.0	4.0	49.8
	Likelihood ratio (−)	0.9	0.8	1.0
	Positive predictive value (PPV) (%)	44.4	13.7	78.8
	Negative predictive value (NPV) (%)	95.2	93.2	96.8
	Accuracy (%)	94.4	94	94.9

**Table 8:** Proposal for laboratory criteria for defining cases of schistosomiasis in ALEs.

Criterion I	Criterion II	Criterion III
Individuals eliminating *S. mansoni *eggs in feces	Positive COPT	Individuals with no *S. mansoni *eggs in feces

With/without positive COPT	Individuals with no *S. mansoni *eggs in feces	Negative COPT

With/without positive ELISA-IgM and/or ELISA-IgG techniques	Without specific treatment in the last 12 months	

	With/without positive ELISA-IgM and/or ELISA-IgG techniques	Positive qPCR-serum and/or positive qPCR-feces
